# Complete Genome Sequence of *Kocuria palustris* MU14/1

**DOI:** 10.1128/genomeA.01195-15

**Published:** 2015-10-15

**Authors:** Michael J. Calcutt, Mark F. Foecking

**Affiliations:** Department of Veterinary Pathobiology, University of Missouri, Columbia, Missouri, USA

## Abstract

Presented here is the first completely assembled genome sequence of *Kocuria palustris*, an actinobacterial species with broad ecological distribution. The single, circular chromosome of *K. palustris* MU14/1 comprises 2,854,447 bp, has a G+C content of 70.5%, and contains a deduced gene set of 2,521 coding sequences.

## GENOME ANNOUNCEMENT

*Kocuria palustris* is one of 18 described species in a lineage that was formerly assigned membership to the genus *Micrococcus* (1). *K. palustris* is an aerobic coccus that was originally recovered from plant rhizoplanes (2), but that has since been isolated from marine environments (3) and more recently from a human patient with keratitis (4). Genomic information of the species is limited to that of a single isolate, *K. palustris* PEL (5), for which a draft genome of 55 contigs is available (GenBank accession no. ANHZ02000000). However, the complete genome of *Microbacterium* sp. KROCY2 (GenBank accession no. NZ_JAGG00000000.1) exhibits a very high level of both nucleotide sequence identity and gene synteny to these contigs.

While characterizing a slowly growing mycoplasmal species, the culture was outgrown by a contaminating isolate, such that the major component of a Pacific Biosciences system-generated genome sequence (performed at the National Center for Genome Resources, Santa Fe, New Mexico) was derived from an actinobacterium. The data from a single single-molecule real-time sequencing (SMRT) cell were assembled using HGAP version 2 (6) resulting in two contigs with >100× coverage and a short overlap. The sequence was permuted to begin with the *dnaA* gene and directly submitted to the National Center for Biotechnology Information for auto-annotation using the PGAP pipeline and recently described annotation strategy (7). The current auto-generated data set comprises 2,521 genes, 2,234 open reading frames, 46 tRNAs, and three rRNA operon sequences (each arranged in a standard 16S to 23S-5S configuration). Analysis of the latter revealed that one of the 16S rRNA sequences is 100% identical to that of the *K. palustris* type strain TAGA27^T^ (2) and the two additional alleles contain the same single nucleotide polymorphism to the reference. The closest phylogenetic relative is *Kocuria halotolerans*; the 16S rRNA genes are 96.8% identical to that of strain YIM 90716^T^ (8).

Comparative analysis of the genome to the 55-contig set from *K. palustris* PEL and to the complete genome of *Microbacterium* sp. KROCY2 revealed extensive similarity and gene synteny. The genome of the latter contains 2,765,822 bp, and is thus approximately 88 kb smaller than that of *K. palustris* MU14/1; the draft genome of *K. palustris* PEL (2,872,112 bp) is approximately 18 kb larger than that of strain MU14/1. In a binary comparison of the two *K. palustris* genomes, major regions of difference are associated with distinct mobile genetic element composition and variably present restriction-modification systems.

The genome sequence is the first to be completely assembled for the species, with the caveat that a complete genome exists for *Microbacterium* sp. KROCY2. Query of the 16S rRNA databases discloses 100% identity between those of this taxon and *K. palustris* TAGA27^T^. Although the source of the inadvertent *K. palustris* MU14/1 inoculant is not known, the data set may be of use in future comparative studies to the genomes of isolates that elaborate the novel antibiotic kocurin (9) or that have been associated with keratitis of the human eye (4).

### Nucleotide sequence accession number.

The complete genome sequence has been deposited at DDBJ/EMBL/GenBank under the accession no. CP012507.
